# Artificial Polyploidy Improves Bacterial Single Cell Genome Recovery

**DOI:** 10.1371/journal.pone.0037387

**Published:** 2012-05-22

**Authors:** Armand E. K. Dichosa, Michael S. Fitzsimons, Chien-Chi Lo, Lea L. Weston, Lara G. Preteska, Jeremy P. Snook, Xiaojing Zhang, Wei Gu, Kim McMurry, Lance D. Green, Patrick S. Chain, J. Chris Detter, Cliff S. Han

**Affiliations:** 1 Bioscience Division, Los Alamos National Laboratory, Los Alamos, New Mexico, United States of America; 2 Department of Energy (DOE) Joint Genome Institute, Los Alamos National Laboratory, Los Alamos, New Mexico, United States of America; Belgian Nuclear Research Centre SCK/CEN, Belgium

## Abstract

**Background:**

Single cell genomics (SCG) is a combination of methods whose goal is to decipher the complete genomic sequence from a single cell and has been applied mostly to organisms with smaller genomes, such as bacteria and archaea. Prior single cell studies showed that a significant portion of a genome could be obtained. However, breakages of genomic DNA and amplification bias have made it very challenging to acquire a complete genome with single cells. We investigated an artificial method to induce polyploidy in *Bacillus subtilis* ATCC 6633 by blocking cell division and have shown that we can significantly improve the performance of genomic sequencing from a single cell.

**Methodology/Principal Findings:**

We inhibited the bacterial cytoskeleton protein FtsZ in *B.* *subtilis* with an FtsZ-inhibiting compound, PC190723, resulting in larger undivided single cells with multiple copies of its genome. qPCR assays of these larger, sorted cells showed higher DNA content, have less amplification bias, and greater genomic recovery than untreated cells.

**Significance:**

The method presented here shows the potential to obtain a nearly complete genome sequence from a single bacterial cell. With millions of uncultured bacterial species in nature, this method holds tremendous promise to provide insight into the genomic novelty of yet-to-be discovered species, and given the temporary effects of artificial polyploidy coupled with the ability to sort and distinguish differences in cell size and genomic DNA content, may allow recovery of specific organisms in addition to their genomes.

## Introduction

Microbial communities are complex ’supra-organisms’ consisting of bacteria, archaea, eukaryotes, and viruses that interact with and depend upon one another in order to survive and thrive in their natural environmental niches. In part due to the intrinsic interdependencies within microbial communities, only a small fraction (<1%) of all bacteria have been cultivated in isolation, a pre-requisite for traditional genome sequencing efforts [1]. While direct shotgun sequencing of environmental communities is possible, assembly of metagenomic data into complete genomes has thus far only been partly achieved for the simplest of microbial communities [2], [3]. Therefore novel approaches need to be devised in order to isolate DNA from these ‘uncultured’ species in sufficient quantities for genome sequencing, if we want to better characterize the microbial world. One method becoming more commonly used to address this problem is the technique of DNA amplification of a single cell’s genome (known as single cell genomics, or SCG) to provide adequate amounts of material for sequencing protocols.

Roger S. Lasken [4] described an approach to SCG through multiple displacement amplification (MDA) [5], [6], , which can generate up to micrograms of DNA starting with as little as femtograms present in a typical bacterium. MDA is based on isothermal (30°C) strand displacement DNA synthesis in which the highly processive φ29 DNA polymerase repeatedly extends random primers on the template as it concurrently displaces previously synthesized copies and exposes single stranded DNA that are used as new templates [8]. The ability to sequence from single cells using amplified DNA was demonstrated by Raghunathan *et* *al*. with flow sorted *Eschericia coli*, *Myxococcus xanthus*, and *Bacillus subtilis* [7]. Whole genome sequencing with amplified DNA has been tested on single cells of several target species such as *E.* *coli* and *Prochlorococcus* [9], and has been used for novel *de novo* sequencing of TM7 [10], [11], flavobacteria [12], [13], and *Prochlorococcus* single cells [14]. These published efforts resulted in fragmented genome assemblies with up to a thousand contigs or more, although extensive and expensive genome closure efforts can reduce this to a much smaller number [13].

A fundamental problem of SCG as presently carried out lies in the fact that only a single copy of genomic DNA serves as the template. Consequently, if that single molecule of DNA is fragmented with double stranded breaks (as can happen during sample preparation), all linking information is lost resulting in a genome sequence gap. When coupled with genome amplification bias (a random bias thought to be introduced by initial random priming events), as well as sequencing bias, the result is an incomplete, fragmented genome. In this study, we sought to overcome this major hurdle by inducing artificial polyploidy in single cells by preventing bacterial cell division (multiple copies of the genome per single cell).

Our approach targets the critical cell division protein FtsZ in such a fashion as to inhibit cell division while maintaining DNA replication. FtsZ is a bacterial GTPase and homolog of mammalian β-tubulin that polymerizes and assembles into a septal ring typically at the middle of a dividing cell to initiate cell division. FtsZ is conserved in almost all eubacterial species and in one kingdom of archaea, Euryarchaeota, but not in Crenarchaeota [15], [16]. The broad phylogenetic distribution of FtsZ makes it an ideal target to inhibit cell division in many different bacterial (and euryarchaeal) species, and may allow the extension of this technique to many previously uncultured and unknown organisms present within environmental samples. Several chemicals have been reported to block the function of FtsZ in various bacteria including *Mycobacterium tuberculosis*, *E.* *coli*, and *Staphylococcus aureus*, typically responding to treatment in filamentous, elongated phenotypes [17], [18], [19], [20], [21], [22], [23], [24], [25], [26]. Here we demonstrate that compound PC190723 can be used to induce artificial polyploidy in *Bacillus subtilis* ATCC 6633 [27]. We present data indicating an increased cell size together with increased DNA content for PC190723-treated cells. We have further demonstrated that single cell genomics after artificial polyploidy induction results in less bias and greater genome coverage than with untreated single cells, and helps improve *de novo* assembly, creating larger and more contiguous sequences.

## Results

### *B*. *subtilis* Responded to Treatment with PC190723 Resulting in Larger Cells

**Figure 1:**
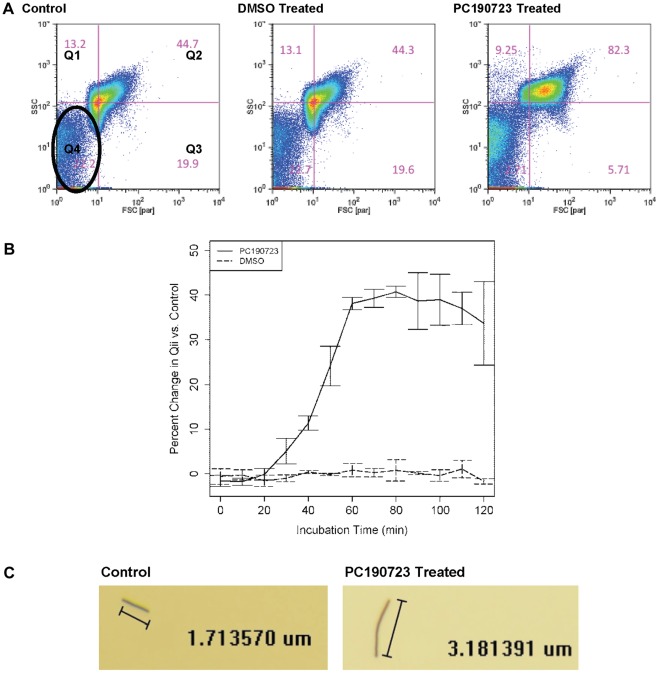
*B. subtilis* response to PC190723 treatment: Increased cell size. (*A*) Typical cytographs from treatments illustrate increase in cell size in PC190723-treated *B. subtilis* versus untreated control and DMSO-treated cells at 60 minutes after treatment. Crosshairs were made at the mode of the untreated control population and applied to the DMSO- and PC190723-treated cytographs. Resultant quadrants give the relative percentage of the population (from 100,000 cells) inhabiting each quadrant. Thus, in quadrant two (Q2) of the control population, 44.7% of the cells are observed, while over 80% are observed in the PC190723-treated population. DMSO has no effect on cell size. Percentages are calculated by omitting the lower left population (*A* Control: black circle) from each cytograph, which likely contains dead cells and cell debris. (*B*) By determining ΔQ2 over time, cell size is shown to plateau after 60 minutes. Error bars are 95% CI. (*C*) Typical light microscope images of sorted untreated control and PC190723-treated cells. PC190723-treated cells are longer than untreated control cells, but the diameter appears unchanged.

We treated *B.* *subtilis* with PC190723 at early exponential phase in liquid culture over different durations of time (see methods). To measure its response to treatment, we used flow cytometry and microscopy to determine changes in cell size when compared to untreated control populations. Cytographic data from flow cytometry showed that PC190723-treated cells became larger, as measured by both side and forward scatter, during the course of treatment compared to untreated control cells (Fig. 1*A*). The core or mode of the cytographs represents the majority of the cell population, often depicted in red. We put a crosshair at the core of the control population (Fig. 1*A* Control) and applied the same setting to the respective DMSO- and PC190723-treated samples resulting in quantified cell distributions in quadrants, which was used as indicators of cell size change (Fig. 1*A, 1B*). Cells in quadrant two (Q2) are generally larger than the ones located in other quadrants (see Methods for details). Briefly, *B.* *subtilis* treated with PC190723 did not show any changes in cell size for the first 20 minutes. At 30 min after treatment, 5% of the treated population showed an increase in cell size, 11% at 40 min, and 24% at 50 min. The change in Q2 plateaued at 60 minutes onwards with an average change of around 40%, and about 80–85% of cell particles located at Q2. When cells were treated with DMSO alone, the solvent of PC190723, no change in cell size was observed.

Exact change in cell size was measured via light microscopy (Fig. 1*C*). Fifty PC190723-treated single cells and 26 control single cells (sorted from the mode of each population) were analyzed showing that the treated cells were 2–3 times longer than the control cells (t-test p<0.0001; Fig. S1). This process also verified that a single cell was deposited from the cell sorter, except for a small portion of the control population. About 15% untreated single particles and 10% PC190723-treated cells were actually doublets butted together. Complete separation of such doublets could not be achieved despite rigorous vortexing.

### *B*. *subtilis* Treated with PC190723 Contained More DNA Per Cell

**Figure 2:**
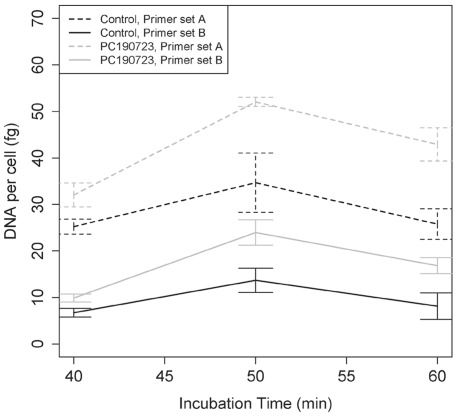
*B. subtilis* response to PC190723 treatment: Increased DNA content. DNA content was assessed for PC190723 treated (maximum effect from ten replicates) and control cells via six replicates of 50 sorted cells for primer sets A (located near the origin) and B (located near the terminus). Theta or multi-fork replication may account for the discrepant reports from both primer sets. Cells treated for 50 minutes had more DNA content than the respective untreated controls. Prolonged drug exposure may account for the reducing DNA content at 60-min treatment as related to DNA degradation. Error bars are 95% CI.

In another cell preparation, we analyzed via flow cytometry ten treatment and control preparations. We sorted 50 cells from the core PC190723-treated and untreated control samples showing the largest Q2 difference at 40-, 50-, and 60-min to estimate DNA content per cell through qPCR assays. *B. subtilis* treated for 50 minutes yielded the highest amount of DNA content, although the 40- and 60-min treated cells also suggested polyploidy (Fig. 2). Primer set A, which amplified a genomic region close to the replication origin (Table S1), reported roughly 50% more DNA, while primer set B, which amplified a region close to the replication terminus, reported 75% more DNA in the 50-min PC190723-treated cells than the untreated controls. Both treated and controls show an elevated amount of DNA relative to the expected amount (approximately 4.5 fg, as the average *B. subtilis* genome size is 4.2 Mbp), which likely relates to stages in the growth cycle. In a more replicated study, we further verified elevated DNA content per cell in 50-min, PC190723-treated *B.* *subtilis* versus untreated controls (Fig. S2).

### PC190723-treated *B. subtilis* Cells Yielded Highest Genomic Coverage with the Least Amplification Bias

**Figure 3:**
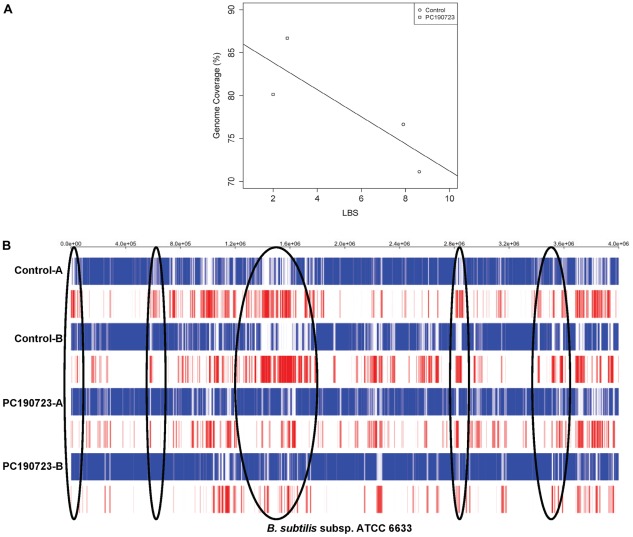
PC190723-treated cells have increased genomic coverage and less amplification bias. (*A*) Genome coverage of mapped reads increases with treatment. Also, LBS correlates with genome coverage (R^2^ = 0.6998, p = 0.1634). Best-fit line determined by least-squares linear regression. (*B*) Mapped to *B. subtilis* ATCC 6633 genome, untreated control cells have more gaps (red lines) than inhibited cells. Similar gap placement on the genome suggests amplification bias. Because of increased genomic template in polyploid cells, the number of gaps is greatly reduced (black circles).

DNA from single cells (N = 14 PC190723-treated and N = 18 untreated candidates from two 96-well PCR plates) was amplified by MDA. Amplification bias for each amplicon was calculated using the locus bias score (LBS) [28], which measures the variation in amplification from MDA across the genome using six qPCR primer pairs (Table S2; *Materials and Methods –* Determining Maximum DNA Quantity Per Treatment Time). To determine the representative MDA candidates for genomic sequencing comparison studies, we selected the top two candidates having the least theoretical amplification bias for each candidate from plate one. Table 1 summarizes the results of the four selected, sequenced candidates. The two PC190723-treated single cell MDA samples used for sequencing had LBS scores of 2.00 and 2.64 (PC190723-A and –B, respectively) and two untreated single cell samples had scores of 7.9 and 8.62 (untreated-A and –B, respectively). All candidates’ sequencing data were normalized to nearly 8.78×10^6^ raw reads and mapped to the *B. subtilis* ATCC 6633 genome (incomplete). PC190723-A had nearly 80.1% genome coverage, while PC190723-B had 86.7%. Untreated-A had 76.7% genome coverage, while untreated-B had 71.1%. On average, there is about 9.5% more genomic coverage gained from the PC190723-treated cells (Fig. 3*A*; best-fit line determined by linear regression). Thus, increased genomic coverage translates to reduced gaps in the mapped genome (Fig. 3*B*). The similar placement of these gaps in the genome across all the four selected candidates suggests amplification bias during the MDA process. However, the presence of these gaps are reduced or eliminated at that specific region in the genome from polyploid cells (Fig. 3*B*, black circles). Across both plates, amplification bias is significantly decreased in the PC190723-treated cells than in the control samples (t-test of square root transformed data, N = 31, p = 0.0087; Fig. S3).

**Table 1:** Summary of candidates selected for sequencing.

Candidates	LBS	% coverage (mapped)^a^	% coverage(*de novo*)^b^
PC190723 -A	2.00	80.1	58.9
PC190723 -B	2.64	86.7	71.6
Control -A	7.90	76.7	54.4
Control -B	8.62	71.1	50.1

a normalized to nearly 8.78×10^6^ raw reads.

b normalized to 1.0×10^7^ raw reads.

### PC190723-treated *B. subtilis* Cells Yielded Better Genome Assemblies

**Figure 4:**
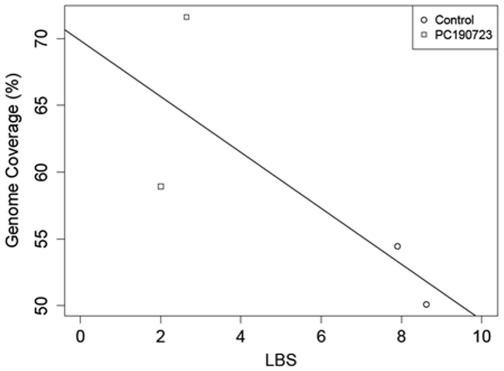
Treated cells have better sequencing results. Genome coverage following *de novo* genome assemblies similarly increases as LBS decreases due to treatment with PC190723 (R^2^ = 0.6033, p = 0.2233). Best-fit line determined by least-squares linear regression.

Mapping raw reads, however, represents only cases in which a reference genome is available. Consequently, we re-analyzed the sequence data and performed *de novo* assemblies with 10 M raw reads each. We found that PC190723-A and -B had 58.9% and 71.6% genome coverage, respectively, while untreated-A and -B had 54.4% and 50.1% genome coverage, respectively (Table 1). Thus, nearly 13% more genomic coverage is gained on average when inhibiting cell division and performing *de novo* genome assemblies (Fig. 4; best-fit line determined by linear regression).

## Discussion

Single cell genomics is a great tool to study genomes of ‘unculturable’ bacterial species, although significant challenges remain to be tackled before it becomes an efficient tool for broad applications. Our results indicate that induced artificial polyploidy will improve the performance of single cell genomics. It is already known that adding more cells to an MDA reaction results in better genome coverage [7]. Average locus representation in DNA amplified from a single cell was shown to be approximately 30%, and increased to near 80% with 10 cells as the starting amplification template. Instead of adding more cells of the same species, which one cannot do easily from a complex environmental or human associated sample, we increased the copies of genomic template within a single cell. This has achieved the same goal of better genome coverage.

By inducing polyploidy, we have shown that the treated cells have higher genomic recovery than the untreated cells, regardless of whether the reads were either mapped to a reference genome (Figs. 3*A and 3B*) or assembled *de novo* (Fig. 4). Both assemblies suggest a trend between genomic coverage and LBS, though the lack of significance is due to the sample size. Inducing polyploidy allows for more even amplification across the genome (Fig. S3, S4), while reducing gaps observed in the genome assemblies (Fig. 3*B*). The similar placement of these gaps along the genome is likely due to amplification bias. However, these gaps are greatly reduced in the polyploid cells. The observed reduction of amplification bias in the mapped genome of polyploid cells gives strong support for the use of the LBS vetting technique as the polyploid MDA candidates were selected for this study based on to their respective LBS values.

We suggest that LBS correlates with genome coverage following sequencing, whether using *de novo* assemblies or mapping reads to a reference genome, though additional data may statistically strengthen this claim. This technique allowed us to choose the best among MDA products for later sequencing and provided support for the superiority of the MDA products of the PC190723-treated cells. The technique provides a means to vet samples before committing to the expense of sequencing samples that could be highly contaminated or would only produce very low coverage due to extreme amplification bias. Because this method requires some prior knowledge of a genome, it would not be useful for sequencing single cells from a completely uncharacterized community. Alternatively, other vetting procedures may be employed which require less or no previous knowledge of genomic content, such as shallow sequencing and identification of conserved single copy genes [13], or verification that frequency versus coverage histograms are normally distributed (i.e., no particular region of the genome has been amplified much more than another part).

The theoretical genome coverage with two to four copies of chromosome should be beyond 90% (Fig. S5). However, our selected polyploid candidates having an estimated two to three chromosome copies yielded 83% mapped genome coverage, on average. Various explanations may account for this less-than-expected performance. First, MDA performance might be related to the stage of chromosomal conditions, such as gene expression, gene-regulator binding events, and sensitivity to nuclease activity. Such chromosomal conditions in individual cells should generally be more different than those of the multiple copies of chromosomes in the same cell. Therefore, the compensation advantage among multiple-copy chromosomes in the same cell could be reduced as they had the same accessibility to MDA. Second, the chromosomal structure in dividing cells, particularly in the induced polyploid cells, might be more compact than that of an untreated, single copy cell [29]. Such compacted structure could, for example, reduce polymerase binding due to restricted template availability. Third, our experiment suggests that compound PC190723 reduces survivability after a period of exposure during growth (Fig. 2). PC190723 and other FtsZ inhibitors are initially intended as options to antibiotics [17], [18], [19], [20], [21], [22], [23], [24], [25], [26], [27], though we have demonstrated through PC190723 that such FtsZ inhibitors can alternatively be used to improve genomic recovery from single cells. It is possible that the DNA in some of the treated cells might begin to degrade at the 50-min treatment time, which may explain the observed decrease in DNA content after 60 minutes of exposure. Fourth, compared to chromosomes from different single cells, the multiple copy chromosomes from the same cell could be more likely co-located together in the MDA process, thereby competing for MDA reagents and resulting in less amplification.

During the progress of our work, a study was published demonstrating that natural polyploidy helps genome assembly [30]. Woyke and colleagues amplified DNA from a single bacterial cell of Candidatus *Sulcia muelleri* DMIN that exhibits natural polyploidy with hundreds of copies of its genome per cell. The 240 kb genome was completely finished with the amplified DNA from a single cell, which is the first and only completely finished single cell genome. In our experiment, the treated single cell has four copies of the genome, at best, based on its doubling time. Compared with the control cell, our qPCR results indicate between two and three copies of the genome. However, in this case the control cell may be elevated artificially due to the occurrence of doublets (i.e., incompletely divided cells that were sorted as a single particle verified via microscopy), which may account for the higher than theoretically possible DNA content per cell for untreated cells of around 4–5 fg. Untreated cells were observed to have a doublet rate of 15%, whereas PC190723-treated cells were seen to have a doublet rate of around 10%. In addition, a portion of the control cells having already replicated their chromosomes, but have not yet divided, may also account for this discrepancy.

Our qPCR results (Fig. 2) show that we recover more DNA content per treated cell than when using primer set A, while primer set B reports significantly less. This observed difference in yield may be due to theta replication, where two replication forks are present in the chromosome (thus, forming a chromosomal structure resembling θ) starting at the replication origin, thereby making more available priming sites for set A. Multi-fork replication may also account for this observation, where a second round of replication begins at the two oriC when the first replication has yet to be completed. This opens, at least, four additional priming sites for primer set A compared to the one for primer set B. Because we find more recovery with primer set A over primer set B respective to the treated and untreated cells, it is very possible that the cells sorted for this assay contained chromosomes in mid-replication.

PC190723 is a known antimicrobial effective against *Bacillus*, *Staphylococcus*, and probably against a range of other Gram-positive bacteria [27]. This indicates that artificial polyploidy can be applied to such broad Gram-positive bacteria that are important players in the human microbiome as well as in natural and agricultural [31], [32], [33] ecosystems. There are a number FtsZ inhibitors available that are effective against Gram-negative or phylogenetically diverse bacteria, which may broaden the applicability of the approach demonstrated in this study [19]. As most FtsZ inhibitors have a different spectrum in blocking bacterial cell division, it is conceivable that a cocktail of inhibitors could be used for a complex environmental sample.

To test the practicality of our method on an environmental sample, we attempted to arrest cell division on a heterotrophic desert consortium. Applying the same methods as described, but using another FtsZ inhibitor, 3-MBA [17] dissolved in LB media, our preliminary data based on cytographic evidence shows a visible shift of the mode of the 3-MBA-treated population toward Q2, with an average of 20% of the heterotrophs responding to treatment (Fig. S6). Although additional studies are needed, this evidence gives a promising outlook for the applicability of our novel method on an environmental community.

It is possible to target other cell division machinery to create artificial polyploidy, such as Min proteins. However, there are no or less chemicals reported blocking functions of those targets than FtsZ. Even some antibiotics that prohibit cell wall synthesis can be tested for this application as well. With a limited study, we observed an increase in cell length under treatment with cephalexin at a low dose (data not show). Other researchers saw the filament formation with other antibiotic treatment as well [34]. Further study is needed to verify if the observed longer cells actually contain more DNA. A combination of these approaches will likely fulfil the promise of artificial polyploidy.

One limitation of our method is that it requires growth of the bacteria in the laboratory, however, it can be done in a community setting, which will allow for the growth of more types of bacteria than could be grown in isolation [35]. Another drawback may be the different growth rates of environment bacteria, which may make it difficult to determine when and for how long to treat a sample. One other limitation is determining the ideal dosage of other FtsZ inhibitors, as the commercial intent of these compounds is an alternative form of antimicrobials to antibiotics. These limitations are not insurmountable, however, and the net benefit of our approach should be the availability of quality genomic data that is not currently attainable through either metagenomics or current SCG methods.

## Materials and Methods

### Blocking and Detecting Cell Division

#### PC190723 stock preparation

PC190723 (Cayman Chemical) was dissolved in 100% DMSO to 5 µg/µl and stored at −20°C.

#### *B. subtilis* preparation

Ten replicates of one *B. subtilis* colony were inoculated in 2 ml no-glycerol LB broth (Teknova, Hollister, CA) and incubated for 11 hr at 37°C while shaking at 200 rpm. Each stock culture was measured for OD_600_, diluted to ∼0.05 OD_600_ in 20 ml LB broth contained in a 50 ml conical Falcon™ tube, and incubated as described earlier until reaching ∼0.1 OD_600_ (approximately 60 min). Five milliters of each 20 ml culture was immediately aliquoted into three 50 ml conical Falcon™ tubes, thereby representing one of three preparation types: (1) Control – as is; (2) DMSO-treated control – addition of 1 µl 100% DMSO, and; (3) PC190723-treated – addition of 1 µl PC190723 stock (1 µg PC190723 final). All preparations were immediately incubated thereafter. Harvested cells were vortexed thoroughly to separate the cells that still linked together after cell division.

#### Determining maximum cell size and treatment time

Three replicates of *B. subtilis* cultures were prepared as previously described, but scaled up to 20 ml aliquots of the three preparation types. One milliliter subsamples were taken at 10 min intervals up to 120 min, stored on ice, vortexed for 5 min at 1,600 rpm, 10^5^ data points were collected via flow cytometry (BD Influx™, BD, Franklin Lakes, NJ), and analyzed for changes in cell size using FlowJo ver. 8.8.6 (Tree Star, Inc., Ashland, OR). Data near the origin were out-gated as they are most likely non-viable cells and cell debris. The collected data were analyzed under logarithmic scale of side scatter (SSC, y-axis) versus forward scatter (FSC, x-axis), where crosshairs were set at the mode of each control cytograph for every time point, thereby creating quadrants and enumerating the relative percentage of cells occupying that quadrant. Because both SSC and FSC are measurements of cell size, it is, therefore, expected that the larger PC190723-treated cells will shift in the direction of quadrant two (Q2) relative to its non-treated control. The numerical value of change in Q2 (ΔQ2) over time is then calculated by subtracting percent population values in the control Q2 from the treated Q2. The differences were plotted over time to determine the increase in cell size in relation to treatment time. In our cell preparations involving ten replications (not including the initial Determining Maximum Cell Size Per Treatment Time study), the sample set having the median ΔQ2 was selected for sorting cells intended for qPCR and MDA assays.

Cell size was also measured using light microscopy. Cells were sorted directly onto microscope slides from the mode of the PC190723-treated and control populations. The samples were heat fixed, stained with crystal violet for 1 min, washed with dI water, and air-dried. Samples were viewed using a Zeiss Axiophot light microscope (Carl Zeiss Inc. Oberkochen, Germany) at 400×. Pictures were taken using an attached Olympus DP71 Camera (Olympus Corporation, Center Valley, PA). Cells were measured using Image Pro Plus ver. 6.2 (Media Cybernetics, Inc., Bethesda, MD).

#### Determining maximum DNA quantity per treatment time

To test if the maximum change in cell size at a particular treatment time interval relates to maximum chromosome copy per cell, qPCR assays of sorted cells were necessary to determine the maximum DNA quantity. Ten replicates of *B. subtilis* cultures were prepared and the subsequent FACS data analyzed for maximum change in cell size as previously described. The PC190723-treated replicate and its respective control showing the most change in cell size at the 40-, 50-, and 60-min sampling intervals (Fig. 1*B*) were selected for this assay. Fifty cells were sorted from the mode of each population into 2 µl 40 mM Tris-HCl buffer pH = 9 in a 96-well PCR plate. DNA amount was determined using qPCR and primer sets amplifying regions of the Bacillus genome near the origin of replication and the replication terminus (Primer sets A and B, respectively). Ten primer sets were designed using Primer [36] (Table S2) and *B. subtilis* str. 168 genome (GenBank NC000964), and validated with single cell qPCR assay. Six of them were selected for sensitivity.

#### qPCR

The qPCR mastermix was prepared as per manufacturer’s protocol using the Power SYBR® Green PCR Master Mix (Applied Biosystems, Carlsbad, CA). We used the 7500 Fast Dx Real-Time PCR Instrument™ (Applied Biosystems, Carlsbad, CA) under the following cycling parameters for all primer sets: One denaturation cycle of 95°C for 5 min, 45 amplification cycles of 95°C for 10 sec, 45°C for 10 sec, and 72°C for 1 min; followed by a melt curve analysis. Final concentrations for the following qPCR mastermix reagents for each 25 µl were as follows: primer mix (IDT, Coralville, IA) to 1 mM; BSA (NEB, Ipswich, MA) to 1.6×; Power SYBR® Green PCR Master Mix (Applied Biosystems, Carlsbad, CA) to 1×. A standard curve was made from each primer set using 1.0, 0.1, 0.01, 0.001, and 0.0001 ng *B. subtilis* DNA extracted using the Ultra Clean Microbial DNA Kit (MoBio Laboratories Inc, Carlsbad, CA).

#### Preparation for MDA and genomic analyses

Ten replicates of *B. subtilis* cultures were prepared, but incubated for 50 min at 37°C while shaking at 200 rpm, and the subsequent FACS data analyzed as previously described. The 50-min treatment replicate having the median ΔQ2 value from all ten preparations was chosen for qPCR and MDA assays, and for subsequent genomic analyses. Single cells from the mode of each population were sorted into 2 µl lysis buffer in a 96-well PCR plate and the DNA was amplified (See Whole Genome Amplification). The amplicons from each MDA reaction were analyzed for genomic amplification biases by calculating its respective locus bias score (LBS; See Determining the Least Biased MDA Amplicon) [28]. Amplicons with the lowest LBS from plate one of two were sequenced on the Illumina® GAIIx™ sequencing platform.

### Flow Cell Sorting

#### Cytometer preparation

FACS analysis and targeted cell sorting were performed on the Influx™ flow cytometer (BD, Franklin Lakes, NJ) using a 488 nm laser at 22 psi at an average rate of 4,000 counts per second (cps) with a 100 µm nozzle. To greatly reduce the introduction of extraneous nucleic acid material, particularly when sorting cells is scheduled, instrument preparatory steps were performed. Prior to analysis and sorting, the sheath and sample lines were flushed with 10% bleach for 30 min followed by sterile dI water and air-dried. The sheath tank was thoroughly cleaned with 10% bleach and UV-exposed to a 254 nm lamp overnight. Pre-sterilized sheath fluid (25 mM HEPES with 100 mM sodium salt; BioSure®, Inc., Grass Valley, CA) was loaded into the sterile sheath tank the following morning and UV-exposed for over 60 min. The sheath fluid was filtered through a 0.22 µm filter during all FACS analyses. The sort chamber was cleaned with 10% bleach and 100% MeOH, where appropriate.

#### Data collection

10^5^ data points per sample were collected using Spigot© ver. 6.1.9 (BD, Franklin Lakes, NJ) and analyzed for increased changes in cell size under forward scatter (FSC) versus side scatter (SSC) in logarithmic scale using FlowJo ver. 8.8.6 (Tree Star, Inc., Ashland, OR). Prior to sorting, calibration procedures were followed as per manufacturer’s protocol. Of importance, the Influx™ was calibrated to deposit targeted cells directly to the bottom center of each 96-well PCR reaction tube, as verified by visual inspection.

### Whole Genome Amplification

#### Contamination minimization

Because of the high likelihood of untargeted amplification, strict preparatory steps were followed to minimize nucleic acid contamination in the laboratory. Summarily, all workspaces and equipment were cleaned with 10% bleach solution. Glassware, disposables, reagents, tools, etc. were autoclaved, stored in a PCR hood, and UV-exposed over 60 min. Workers wore disposable lab coats, masks (covering nose and mouth), and were double-gloved during all procedures. Two HEPA filtration units (BlueAir 650 E; Chicago, IL) cycled the air of our closed-door laboratory at least six times before any work commenced. Air filtration remained in operation throughout sorting and MDA procedures.

#### Lysis buffer preparation

Alkaline cell lysis buffer was prepared and quality tested at least one day prior to our study [37]. DI water was autoclaved (120°C at ∼17 lb/in^2^ for 60 min) in a 50 ml Corning® jar, stored in a PCR hood, and UV-treated for 60 min. The sterilized dI water was added to DTT (50 mM final concentration; Acros Organics, Thermo Fisher Scientific, Waltham, MA), which was pre-measured into sterile 50 ml conical Falcon™ tubes in a PCR hood, followed by the addition of 8 M KOH (Hach Chemicals, Loveland, CO) to final concentration of 200 mM. The tube was sealed, vortexed, and the lysis buffer was vacuum-filtered through a 0.22 µm screen. The lysis buffer was stored at 4°C in foil. Lysis buffers stored longer than three weeks were discarded.

#### Sorting cells for MDA

In the PCR hood, 2 µl of freshly prepared lysis buffer was aliquoted into a 96-well PCR plate, sealed with an adhesive plate seal, and briefly centrifuged. Only the intended reaction wells were exposed during the sort process. Immediately after cell sorting, the plate was resealed and centrifuged for 30 sec at 1,300 g at 4°C. The reaction plate was stored on ice until the next targeted sorting was performed. Following sorting the plate was kept at −70°C for at least 60 min to ensure proper lysis of sorted cells.

#### Multiple-strand displacement amplification

Thermalcyclers were preheated to and maintained at 65°C. The 96-well PCR plate containing the lysed cells was incubated at 65°C for 10 min to denature the DNA. This modified alkaline lysis process with freezing and heating lysed both treated and untreated *B. subtilis* cell equally well (Figure S7). The 96-well PCR plate was then centrifuged for 30 sec at 1,300 g at 4°C and immediately stored on ice thereafter. The MDA mastermix and reaction plate steps were prepared in a PCR hood at the following final concentrations to a 20 µl final volume, in order: Autoclaved dI water; 10×Φ29 reaction buffer (NEB) to 1×; 500 µM random hexamers (Keck) to 5 µM; 10 mM dNTP mix (Roche) to 1 mM; and 10 U Φ29 DNA polymerase (NEB) to 0.25 U. The MDA mastermix was gently vortexed, centrifuged, and stored on ice until needed. Eighteen microliters were aliquoted into each denatured sample, the plate immediately sealed with an autoclaved 96-well rubber plate sealer, and centrifuged for 30 sec at 1,300 g at 4°C. Genomic amplification was performed at 30°C for 16 hr followed by Φ29 deactivation at 65°C for 10 min, and stored in 4°C. Amplification was visualized via gel electrophoresis using 3 µl of each MDA product. MDA positive controls included 1 ng *B. subtilis* extracted DNA, while negative controls included dI water of the same volume.

#### Determining the least biased MDA amplification

To identify DNA with the least amount of amplification bias, we utilized the locus bias score (LBS) method. This method measures change in representation of various location in a genome following MDA as compared to an unamplified DNA control. Five microliters of 1.5 kb sheared MDA fragments were used as template (E210 Covaris, Woburn, MA). Six primer sets (A-F, Table S2) were used to calculate the LBS for each MDA product using qPCR. MDA negative controls were likewise sheared and assayed to determine contamination levels during the MDA process. No-template qPCR controls were also assayed to ensure the absence of false amplification. MDA amplicons were considered candidates for sequencing if all six primer sets showed amplification of the correct product as verified by melt curve analysis. Of the MDA candidates satisfying these criteria, two of the MDA products having the lowest LBS value from plate one of two were selected for genomic sequencing on the Illumina® GAIIx™ platform.

### DNA Sequencing and Analysis

The Illumina® reads (69 nucleotides) were aligned by BWA [38] with default parameters to *Bacillus subtilis* ATCC 6633 (GenBank accession ADGS00000000) for each sample. The average depth of coverage and percent genome recovery were calculated from alignment results using perl scripts (available upon request). The aligned results were piped to SAMtools ver 0.1.14 [39] for conversion of BWA output format to BAM format and to perform single nucleotide polymorphism (SNP) analysis. The above analysis was also performed with normalized data by the fewest reads.

The Velvet ver. 1.1.02 [40] assembler was used to perform *de novo* assembly, using multiple k-mers and the assembly with best N50 and total contigs size was assembled with different coverage cut-off thresholds. The assembly with the largest contig was initially selected and the unique contigs from all the other assemblies were combined with this initial set. The fraction genome recovered by this unique set of contigs was calculated based on comparisons with the *Bacillus subtilis* ATCC 6633 reference genome using MUMmer [41] (“–maxmatch” parameter).

### Data Analysis

All data were analyzed and figures produced in R (v 2.13) [42]. T-tests and ANOVA were performed on data tested for normality and equality of variance. The ANOVA analysis was performed using the command ANOVA in package Car using type III sum of squares [43]. ANOVA for analyzing DNA amounts per cell (Figure 2) was based on the linear model (DNA content∼Preparation+Treatment+Primer Set+Preparation:Treatment with preparation as a random factor). An F-ratio or p-value is not reported for Preparation as there is no exact solution available for the main effect of a random variable in a three way ANOVA. The effect of treatment for each primer set was calculated using planned comparisons [44].

## Supporting Information

Figure S1 **Effect of PC190723 treatment on cells size.** PC190723 treated cells are significantly larger than untreated control cells (t-test, p<0.0001). Error bars are 95% CI.
(TIF)

Figure S2 **Increased DNA content in 50-min PC190723-treated cells.** In a more replicated study we verified that PC190723-treated cells showed greater DNA content at 50 minutes of treatment. Here, we again started with ten populations, but chose the treated population showing the median effect size for qPCR analysis. Results with standard error are reported in Table S1 **.** In this case there are two separate preparations with six replicate PC190723-treated and control 50-cell sorts for qPCR using primer sets A and B. PC190723-treated cells showed almost two and almost three times the amount of DNA compared to control cells for primer sets A and B, respectively. Separate primer sets are compared using planned comparisons within an ANOVA framework (Table S1). Significance values for comparisons of PC190723-treated and control for primers sets A and B are p<0.001 and p = 0.0125, respectively. Overall significance of the treatment effect is also highly significant (p<0.001).
(TIF)

Figure S3 **PC190723-treated cells have less amplification bias.** LBS results for PC190723-treated and control cells (14 and 18 data points, respectively) suggest that induced polyploidy favors more even genome amplification and thus, less bias. Seventeen samples were omitted from the analysis as one or more primer sets showed either no amplification or amplification of a product with a different melt curve. Error bars are standard deviation. T-test of square root transformed data show treated and control cells to be significantly different (p = 0.0087).
(TIF)

Figure S4 **PC190723-treated cells have reduced amplification bias and more even coverage than untreated control cells.**
(TIF)

Figure S5 **Genome coverage increases and amplification bias decreases as template cell number increases.** To address the possible relationships between template cell number, genome coverage, and amplification bias, we sorted replicates of *E.* *coli* ATCC 29425 in specific numbers for whole genome amplification by MDA and calculated the amplification bias via LBS. The two lowest LBS candidates from each cell number were selected for genome sequencing and subsequent genome assembly (mapped). This study shows that at an additional 20% of the genome is recovered when two templates are used in MDA, compared to that of single cell templates. Having four to eight templates reaches near-complete genomic recovery, and plateus with additional template. Thus, having more than eight starting genomic template will not yield additional genomic information. Concurrent with increasing cell number is the decline in amplification bias. We concluded from this study: (1) two copies of chromosomal template will greatly increase genomic recovery, compared to a single template; (2) four to eight template copies is sufficient to obtain near-complete (if not, complete) genome coverage, and; (3) amplification bias reduces with increasing template number. Based on these findings, we expected polyploid *B.* *subtilis* to yield at least 90% genomic coverage based on our qPCR estimates of three chromosomes per cell. “AmpDNA” is 1 ng of purified *E.* *coli* ATCC 29425 DNA amplified via MDA. “unAmpDNA” is 10 µg of purified *E.* *coli* ATCC 29425 DNA used for direct Illumina® GAIIx library preparation. LBS was determined using six sets of *E.* *coli*-specific primers, as previously described.
(TIF)

Figure S6 **Increased cell size in a heterogeneous community.** Heterotrophs from a desert soil consortium were grown in LB and treated with FtsZ inhibitor 3-MBA. After incubating for 120 min, 20% (averaged from two treatment replicates A and B) of the population increased in size compared to the untreated control. No response was observed in the LB-treated control. The modes of each treated population shows a slight shift toward Q2. This preliminary evidence demonstrates the promising applicability of our method of arrested cytokinesis for the purpose of improved genomic recovery from an environmental sample.
(TIF)

Figure S7 **Lysis efficiency.** To test the efficiency of our lysis buffer and procedure, we treated *B.* *subtilis* with PC190723, as described in Methods, incubated for 50 min after treatment, and compared to untreated control grown for the same duration with a lysis experiment. For comparison to treatment with lysis buffer, the untreated control was also sorted into 2 µl sterile water. Twenty cells from each condition were sorted into 2 µl of our lysis buffer at six replicates each in a 96-well plate and had undergone our standard procedure for cell lysis. The entire lysate volume was transferred onto a microscope slide, air-dried, heat-fixed, and simple-stained with crystal violet. Excess stain was gently washed off with sterile dI water. In comparison to the lysis experiment and for verification that cells were sorted into the plate, 20 cells from each cell treatment were sorted directly onto a microscope slide and stained as described above. Our microscopy analysis is based on our direct observation for the presence of intact cells on the microscope slide, thereby comparing the presence of intact *B.* *subtilis* cells before and after lysis. After obtaining the average number of observed cells across the six replicates for each condition, we calculated “% lysis efficiency” as: % LE = 100*(1 - (# Avg. post-lysis cells/# Avg. pre-lysis cells), to take into account for the possibility that some cells may have been washed off the slide during the staining procedure.
(TIF)

Table S1 **ANOVA for DNA content of PC190723-treated and controls cells.**
(DOC)

Table S2 **Primer sets used for calculating LBS and DNA amount.**
(DOC)
