# Supplementary figures and images for: Artificial Polyploidy Improves Bacterial Single Cell Genome Recovery
Source: PLoS One. 2012 May 22;7(5):e37387. doi: 10.1371/journal.pone.0037387 (PMC3359284; doi:10.1371/journal.pone.0037387)

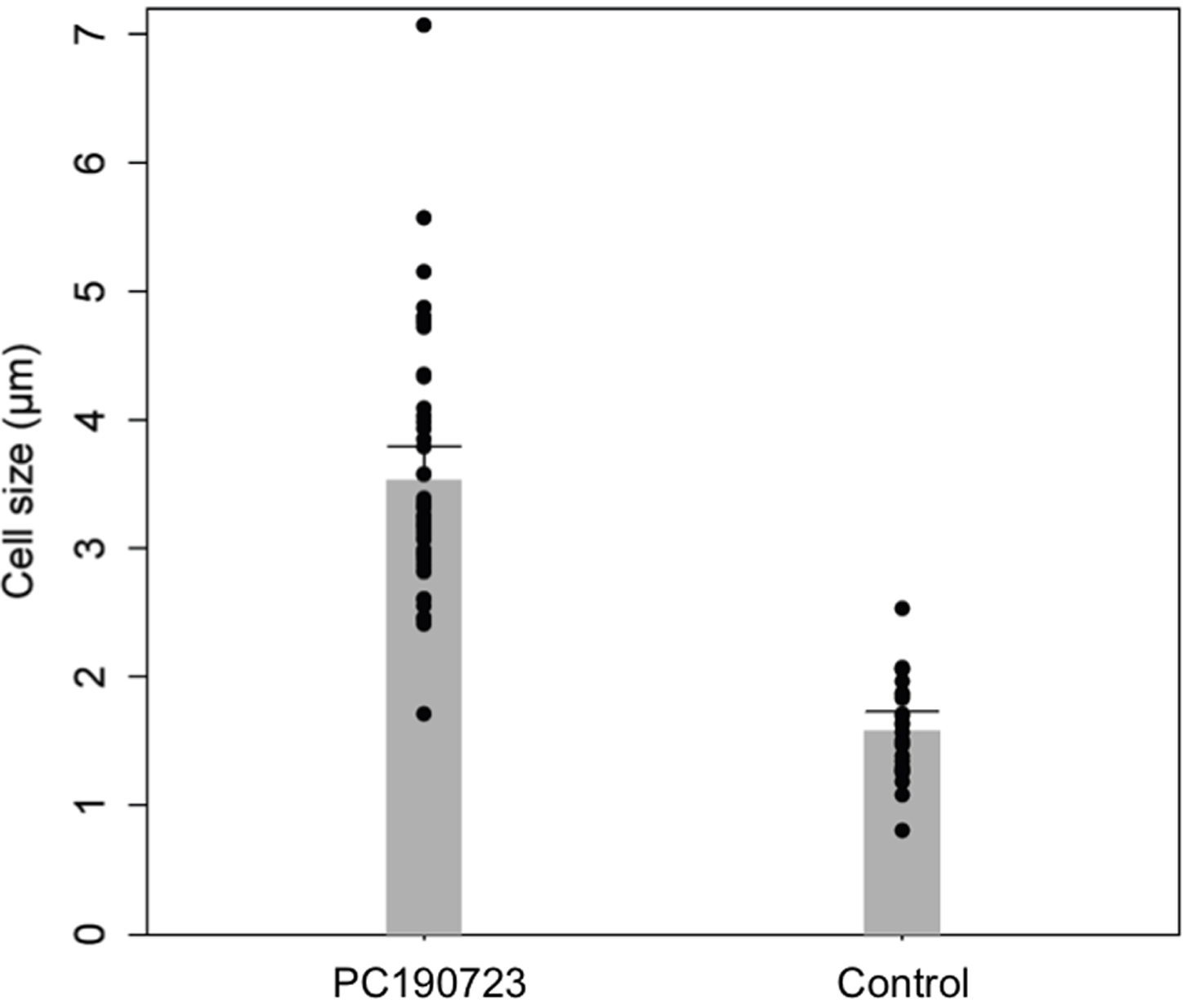

Supplement: Figure S1 — Effect of PC190723 treatment on cells size. PC190723 treated cells are significantly larger than untreated control cells (t-test, p<0.0001). Error bars are 95% CI. (TIF) [file pone.0037387.s001.tif]

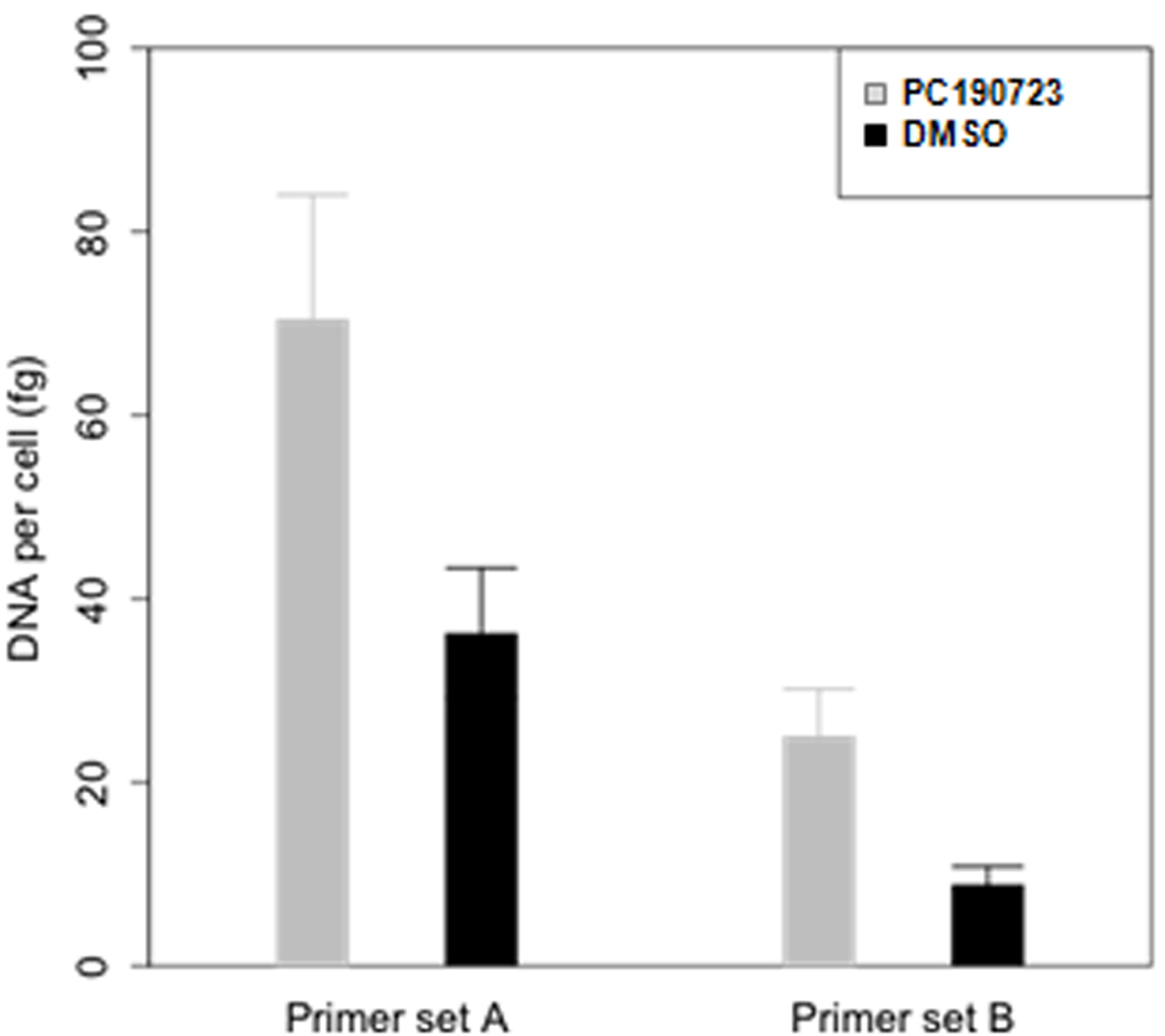

Supplement: Figure S2 — Increased DNA content in 50-min PC190723-treated cells. In a more replicated study we verified that PC190723-treated cells showed greater DNA content at 50 minutes of treatment. Here, we again started with ten populations, but chose the treated population showing the median effect size for qPCR analysis. Results with standard error are reported in Table S1 . In this case there are two separate preparations with six replicate PC190723-treated and control 50-cell sorts for qPCR using primer sets A and B. PC190723-treated cells showed almost two and almost three times the amount of DNA compared to control cells for primer sets A and B, respectively. Separate primer sets are compared using planned comparisons within an ANOVA framework (Table S1). Significance values for comparisons of PC190723-treated and control for primers sets A and B are p<0.001 and p = 0.0125, respectively. Overall significance of the treatment effect is also highly significant (p<0.001). (TIF) [file pone.0037387.s002.tif]

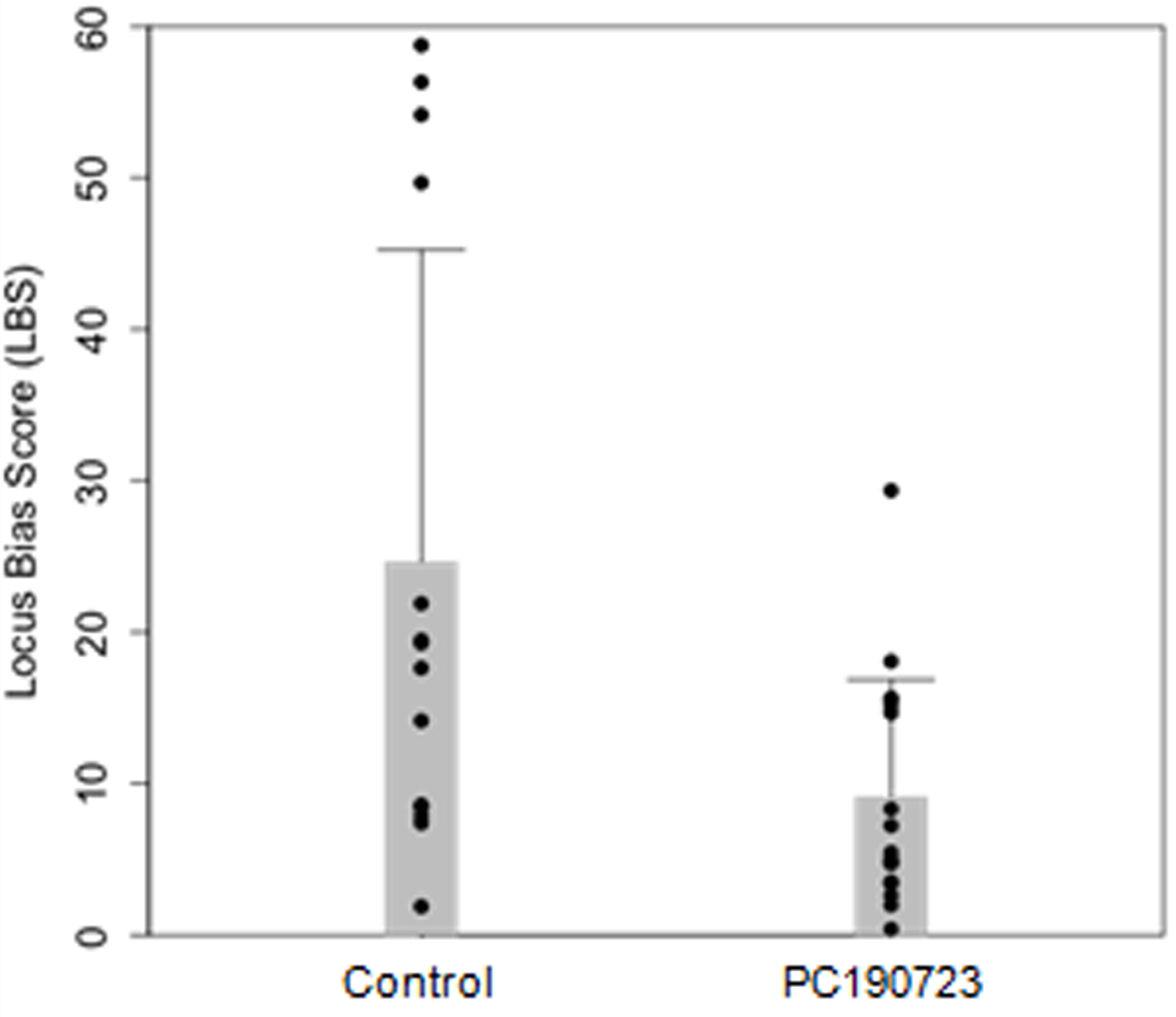

Supplement: Figure S3 — PC190723-treated cells have less amplification bias. LBS results for PC190723-treated and control cells (14 and 18 data points, respectively) suggest that induced polyploidy favors more even genome amplification and thus, less bias. Seventeen samples were omitted from the analysis as one or more primer sets showed either no amplification or amplification of a product with a different melt curve. Error bars are standard deviation. T-test of square root transformed data show treated and control cells to be significantly different (p = 0.0087). (TIF) [file pone.0037387.s003.tif]

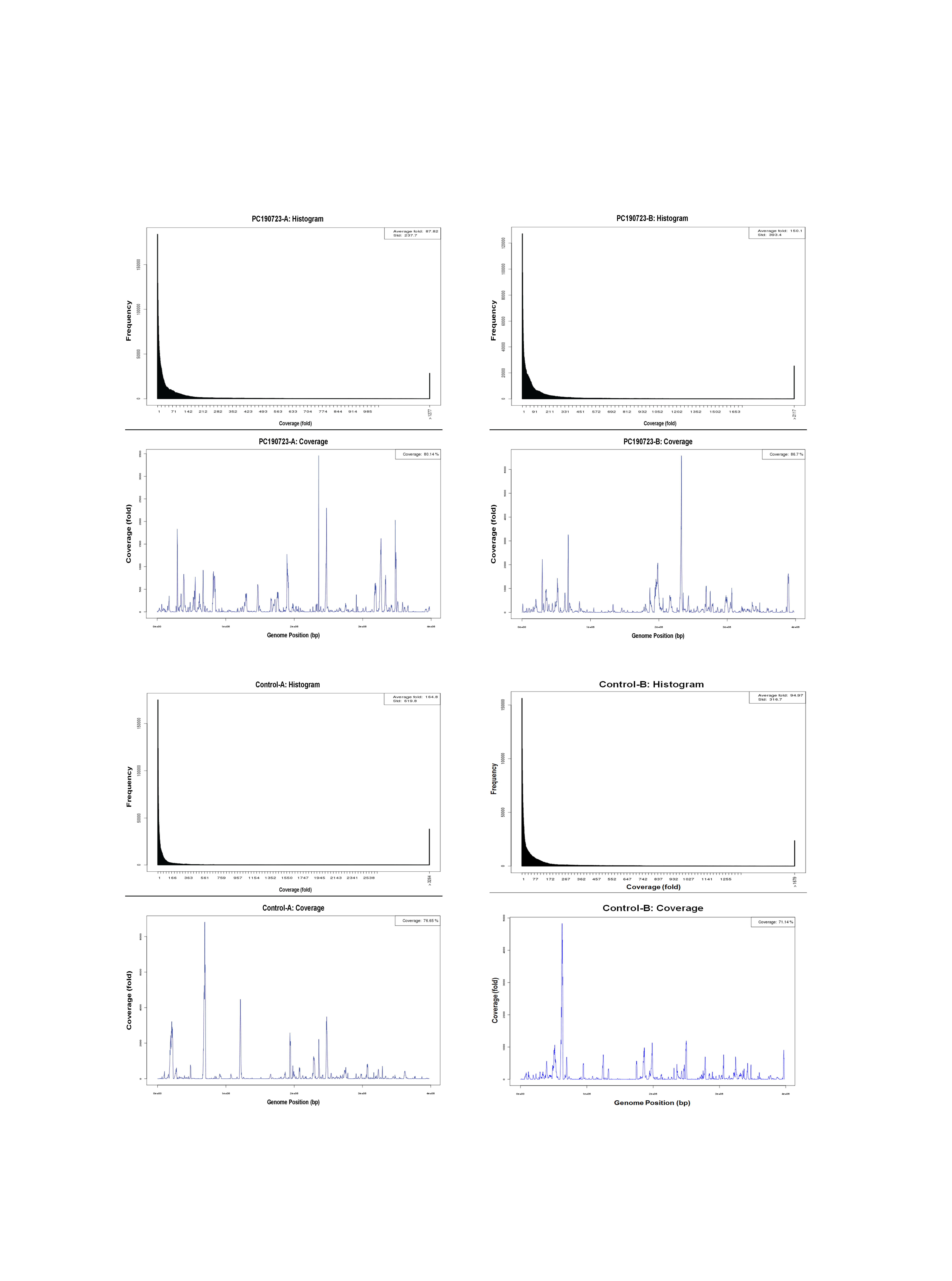

Supplement: Figure S4 — PC190723-treated cells have reduced amplification bias and more even coverage than untreated control cells. (TIF) [file pone.0037387.s004.tif]

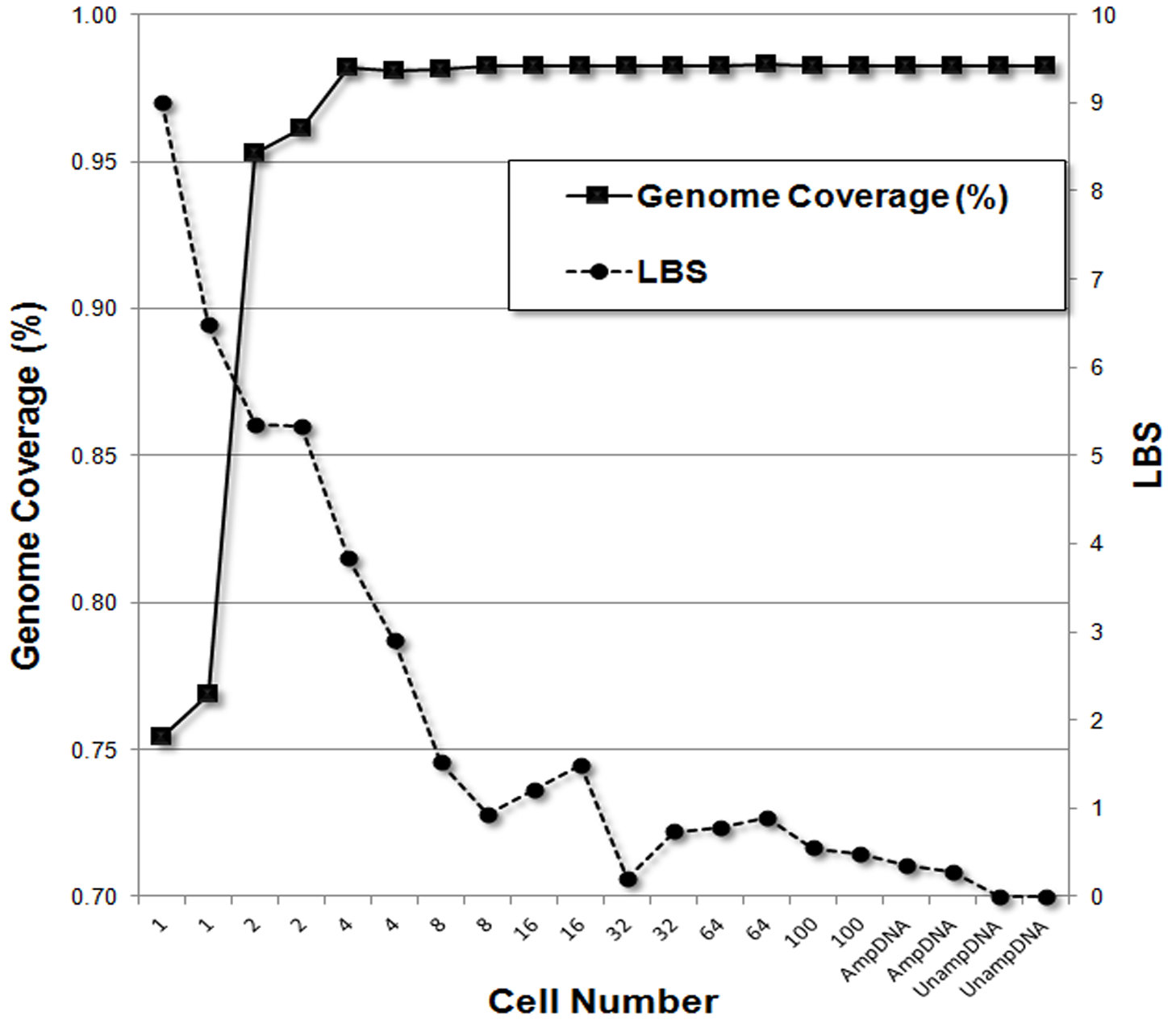

Supplement: Figure S5 — Genome coverage increases and amplification bias decreases as template cell number increases. To address the possible relationships between template cell number, genome coverage, and amplification bias, we sorted replicates of E. coli ATCC 29425 in specific numbers for whole genome amplification by MDA and calculated the amplification bias via LBS. The two lowest LBS candidates from each cell number were selected for genome sequencing and subsequent genome assembly (mapped). This study shows that at an additional 20% of the genome is recovered when two templates are used in MDA, compared to that of single cell templates. Having four to eight templates reaches near-complete genomic recovery, and plateus with additional template. Thus, having more than eight starting genomic template will not yield additional genomic information. Concurrent with increasing cell number is the decline in amplification bias. We concluded from this study: (1) two copies of chromosomal template will greatly increase genomic recovery, compared to a single template; (2) four to eight template copies is sufficient to obtain near-complete (if not, complete) genome coverage, and; (3) amplification bias reduces with increasing template number. Based on these findings, we expected polyploid B. subtilis to yield at least 90% genomic coverage based on our qPCR estimates of three chromosomes per cell. “AmpDNA” is 1 ng of purified E. coli ATCC 29425 DNA amplified via MDA. “unAmpDNA” is 10 µg of purified E. coli ATCC 29425 DNA used for direct Illumina® GAIIx library preparation. LBS was determined using six sets of E. coli-specific primers, as previously described. (TIF) [file pone.0037387.s005.tif]

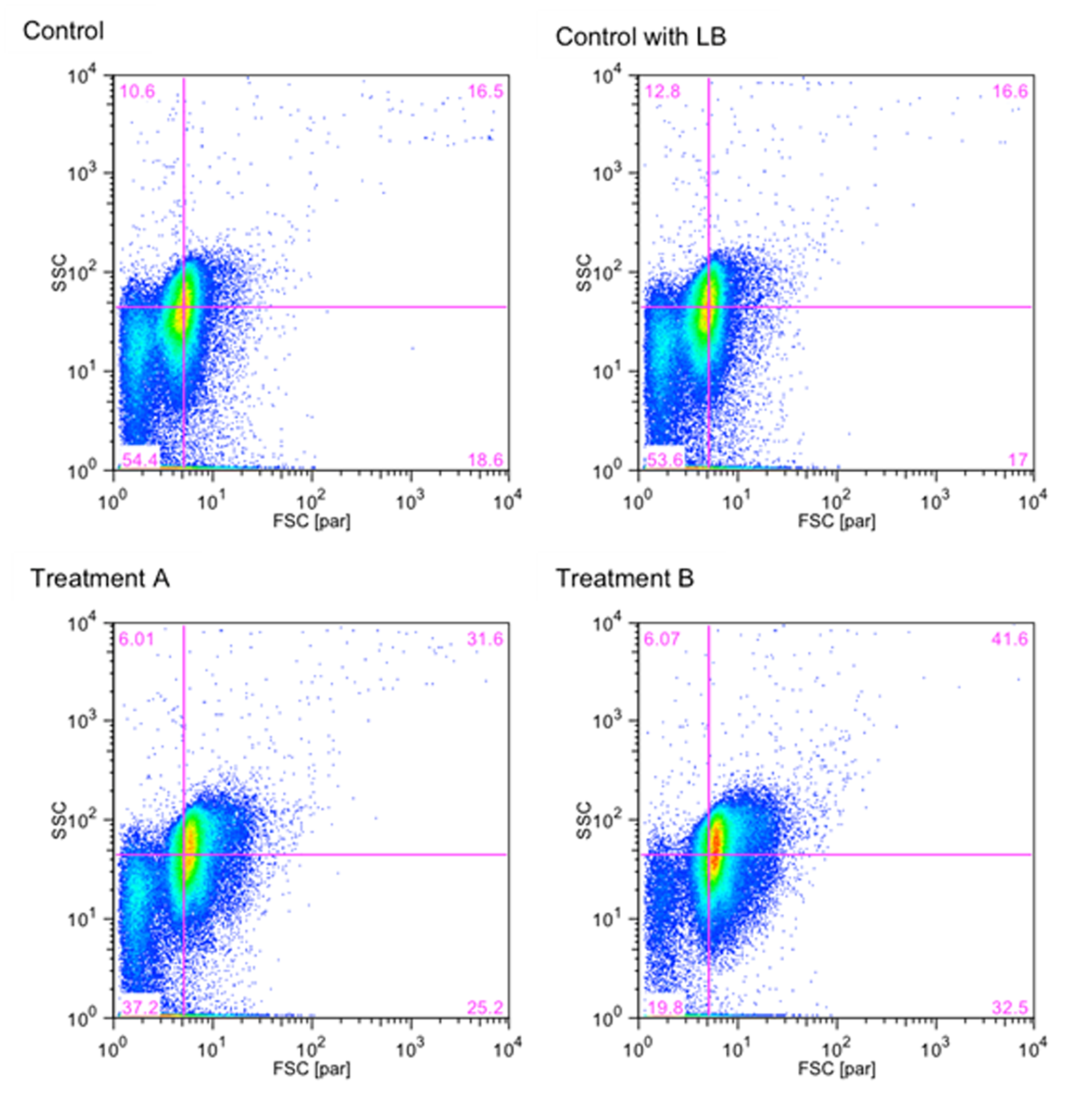

Supplement: Figure S6 — Increased cell size in a heterogeneous community. Heterotrophs from a desert soil consortium were grown in LB and treated with FtsZ inhibitor 3-MBA. After incubating for 120 min, 20% (averaged from two treatment replicates A and B) of the population increased in size compared to the untreated control. No response was observed in the LB-treated control. The modes of each treated population shows a slight shift toward Q2. This preliminary evidence demonstrates the promising applicability of our method of arrested cytokinesis for the purpose of improved genomic recovery from an environmental sample. (TIF) [file pone.0037387.s006.tif]

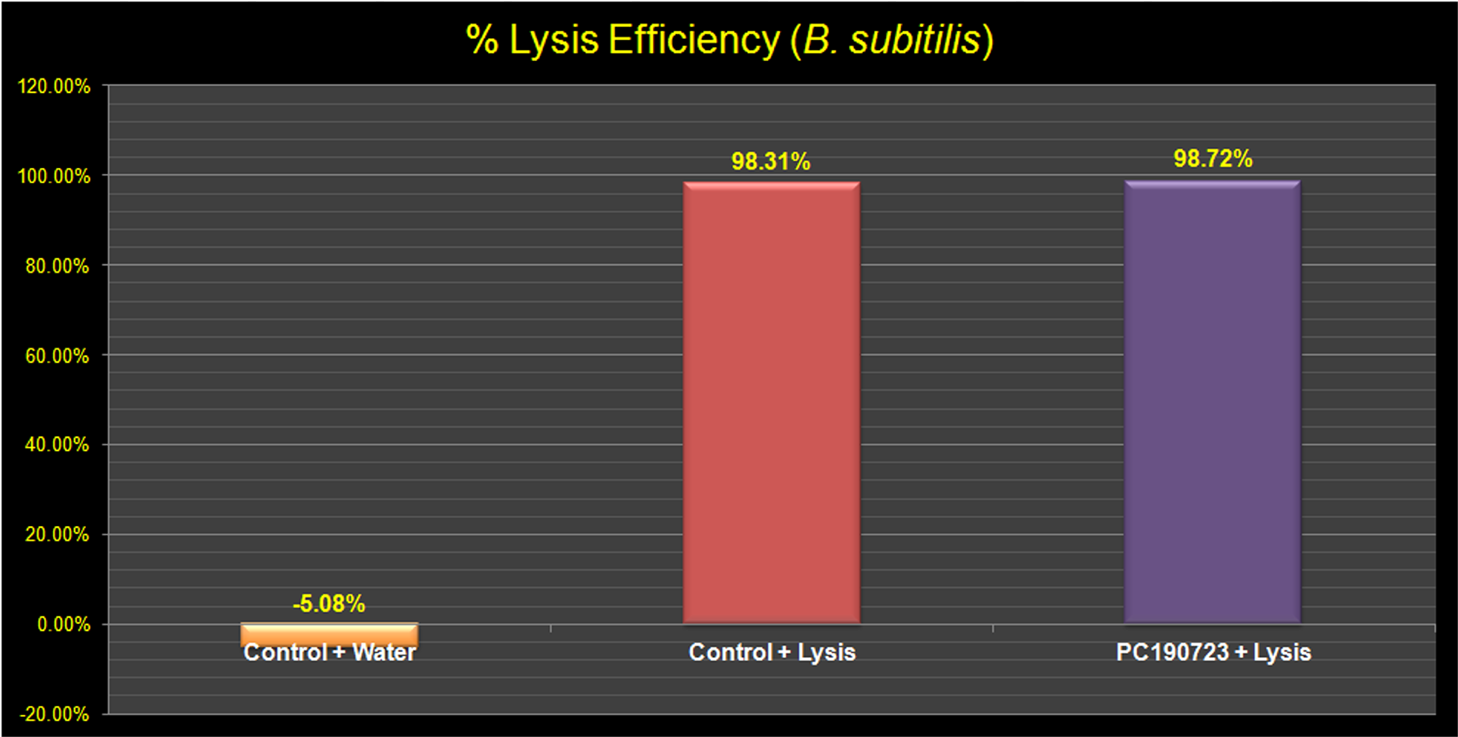

Supplement: Figure S7 — Lysis efficiency. To test the efficiency of our lysis buffer and procedure, we treated B. subtilis with PC190723, as described in Methods, incubated for 50 min after treatment, and compared to untreated control grown for the same duration with a lysis experiment. For comparison to treatment with lysis buffer, the untreated control was also sorted into 2 µl sterile water. Twenty cells from each condition were sorted into 2 µl of our lysis buffer at six replicates each in a 96-well plate and had undergone our standard procedure for cell lysis. The entire lysate volume was transferred onto a microscope slide, air-dried, heat-fixed, and simple-stained with crystal violet. Excess stain was gently washed off with sterile dI water. In comparison to the lysis experiment and for verification that cells were sorted into the plate, 20 cells from each cell treatment were sorted directly onto a microscope slide and stained as described above. Our microscopy analysis is based on our direct observation for the presence of intact cells on the microscope slide, thereby comparing the presence of intact B. subtilis cells before and after lysis. After obtaining the average number of observed cells across the six replicates for each condition, we calculated “% lysis efficiency” as: % LE = 100*(1 - (# Avg. post-lysis cells/# Avg. pre-lysis cells), to take into account for the possibility that some cells may have been washed off the slide during the staining procedure. (TIF) [file pone.0037387.s007.tif]
