# Supplementary material for: Artificial Polyploidy Improves Bacterial Single Cell Genome Recovery
Source: PLoS One. 2012 May 22;7(5):e37387. doi: 10.1371/journal.pone.0037387 (PMC3359284; doi:10.1371/journal.pone.0037387)
Supplement: Table S1 — ANOVA for DNA content of PC190723-treated and controls cells. (DOC) [file pone.0037387.s008.doc]

Table S1. ANOVA for DNA content of PC190723-treated and controls cells

| **Model Terms** | **df** | **SS** | **F-Ratio** | **p-value** |
| --- | --- | --- | --- | --- |
| Preparation | 1 | 3300.8 | NA | NA |
| Treatment | 1 | 7853.3 | 41.539 | 2.01E-07 |
| Primer Set | 1 | 70.441 | 70.441 | 6.72E-10 |
| Preparation x Treatment | 1 | 2106.4 | 11.141 | 0.00202 |
| Error | 35 | 6617.1 |  |  |

F-ratios and p-values are not reported for Preparation main effect because there is no exact solution available for random factors in a three-way ANOVA
