# Supplementary material for: Artificial Polyploidy Improves Bacterial Single Cell Genome Recovery
Source: PLoS One. 2012 May 22;7(5):e37387. doi: 10.1371/journal.pone.0037387 (PMC3359284; doi:10.1371/journal.pone.0037387)
Supplement: Table S2 — Primer sets used for calculating LBS and DNA amount. (DOC) [file pone.0037387.s009.doc]

Table S2. Primer sets used for calculating LBS and DNA amount

| **Primer Set** | **Sequence (5' to 3')** | **Genome Location (bp from origin)** | **Amplicon Size (bp)** |
| --- | --- | --- | --- |
| A | F- ACAGACGCTGCAAATCTCCT | 3923573 | 82 |
|  | R- GCTGACAATCAACGAGACGA |  |  |
| B | F- AAGCACTGGACGGAGAAAGA | 2168626 | 81 |
|  | R- AGACAGCCCCAAATCATCAC |  |  |
| C | F- GCTTGCGCCTTTAGACAATC | 742845 | 116 |
|  | R- GCTTGCGCCTTTAGACAATC |  |  |
| D | F- CGACCATCTTTGTGGATGTG | 1358646 | 103 |
|  | R- AGCGCCTGTTCTTCTGATGT |  |  |
| E | F- AAATAGTGTGCTCGCCTGCT | 3054950 | 106 |
|  | R- AAGTGGTCGGGAAACTTGTG |  |  |
| F | F- CCTGAATGAACTGCTGACGA | 3434828 | 82 |
|  | R- TCTTTCTGGCGTCTTCACCT |  |  |

*B. subtilis*-specific primer sets were designed and validated for sensitivity in qPCR assays involving DNA quantification of whole cells and post-genome amplification LBS calculations.
